# Fibroblasts: Immunomodulatory factors in refractory diabetic wound healing

**DOI:** 10.3389/fimmu.2022.918223

**Published:** 2022-08-05

**Authors:** Ye Liu, Yiqiu Liu, Wenjie He, Xingrui Mu, Xingqian Wu, Junyu Deng, Xuqiang Nie

**Affiliations:** ^1^ College of Pharmacy, Zunyi Medical University, Zunyi, China; ^2^ Key Laboratory of Basic Pharmacology of Ministry of Education and Joint International Research Laboratory of Ethnomedicine of Ministry of Education, Zunyi, China

**Keywords:** fibroblasts, diabetic foot ulcers, inflammation, fibrosis, infection

## Abstract

Diabetes is a systemic disease in which patients with diabetes may develop peripheral neuropathy of the lower extremities and peripheral vascular disease due to long-term continuous exposure to high glucose. Delayed wound healing in diabetes is one of the major complications of diabetes. Slow wound healing in diabetic patients is associated with high glucose toxicity. When the condition deteriorates, the patient needs to be amputated, which seriously affects the quality of life and even endangers the life of the patient. In general, the delayed healing of diabetes wound is due to the lack of chemokines, abnormal inflammatory response, lack of angiogenesis and epithelial formation, and fibroblast dysfunction. The incidence of several chronic debilitating conditions is increasing in patients with diabetes, such as chronic renal insufficiency, heart failure, and hepatic insufficiency. Fibrosis is an inappropriate deposition of extracellular matrix (ECM) proteins. It is common in diabetic patients causing organ dysfunction. The fibrotic mechanism of diabetic fibroblasts may involve direct activation of permanent fibroblasts. It may also involve the degeneration of fibers after hyperglycemia stimulates immune cells, vascular cells, or organ-specific parenchymal cells. Numerous studies confirm that fibroblasts play an essential role in treating diabetes and its complications. The primary function of fibroblasts in wound healing is to construct and reshape the ECM. Nowadays, with the widespread use of single-cell RNA sequencing (scRNA-seq), an increasing number of studies have found that fibroblasts have become the critical immune sentinel cells, which can detect not only the activation and regulation of immune response but also the molecular pattern related to the injury. By exploring the heterogeneity and functional changes of fibroblasts in diabetes, the manuscript discusses that fibroblasts may be used as immunomodulatory factors in refractory diabetic wound healing, providing new ideas for the treatment of refractory diabetic wound healing.

## Introduction

Nowadays, diabetes mellitus has become one of the most common chronic diseases. According to the latest literature, more than 500 million people worldwide suffer from diabetes, which means that more than one in 10 adults worldwide suffer from the disease. In 2021, the prevalence rate of diabetes among people aged 20–79 years old in the world was 10.5% (about 536.6 million people), and it will rise to 12.2% in 2045. Research shows that global diabetes-related health spending reached about $966 billion in 2021 and is expected to reach $1,054 billion by 2045 (1). There is general evidence that diabetes represents a significant global burden of chronic conditions in an aging society. In 2019, it was estimated that 19.3% of people aged 65–99 had diabetes. Regarding regional distribution, the countries with enormous numbers of people over 65 with diabetes are China, the United States of America, and India (2). Complications resulting from diabetes can result in high treatment costs, even disability, and reduced life expectancy (3). Based on the type of diabetes, onset time, and degree of control of metabolism, different complications of diabetes can be caused, and chronic diabetic skin ulcer is one of them. Epidemiological studies have demonstrated that diabetic foot ulcers (DFUs) are considered a marker of high mortality in patients with diabetes. Within 5 years after amputation, the mortality rate is as high as 39%–68% (4). Extensive epidemiologic, clinical, and biological studies have reported that half of all patients with DFUs die within 5 years (5, 6).

Typical wound healing processes are hemostasis, inflammation, proliferation, and remodeling. However, the hyperglycemic environment can affect various processes of wound healing. According to reports, patients with diabetes will have a hypercoagulable state and skin function decline in the process of hemostasis (7). Recently, research demonstrated that sensory nerve endings release neuropeptides to inhibit inflammation and promote epithelial wound healing. At the same time, the resident immune cells provide neurotrophic factors for nerve cells and growth factors for epithelial cells. However, hyperglycemia essentially disrupts these interdependencies, leading to inhibited epithelial proliferation, sensory neuropathy, and decreased dendritic cell density, which causes significant delays in wound healing and sensory nerve repair (8). Although the pathogenesis of T1DM is different from that of T2DM, hyperglycemia is a common feature of diabetes, and low-grade inflammation is a potential mechanism for diabetic complications (9). In the inflammatory process, the imbalance between inflammatory factors and growth factors in diabetic patients is the cause of the chronic inflammatory reaction of the wound (10). Studies have shown that the decrease of neutrophil function also affects the susceptibility of wound surfaces in diabetic patients (11). Multiple reports have demonstrated that people with diabetes are at a greater risk of infection-related death (12, 13). Because of the long-term high glucose environment, the migration and proliferation of keratinocytes in patients with diabetes are reduced. This leads to insufficient re-epithelization of the wound and seriously affects the wound healing process (14). Differential expression of extracellular matrix production and reshaping under the influence of fibroblasts leads to poor wound healing in diabetic patients (15). Biofilm bacteria are prevalent in patients with DFUs. The bacteria develop resistance to antibiotics. Studies have shown that infection increases the time to wound healing and the possibility of lower extremity amputation. Despite antibiotics, amputations occurred in 24.5% of patients with DFUs (16).

Fibroblasts are different from other cells such as epithelial and endothelial cells due to their unique spindle-shaped morphology. Fibroblasts are prominent in the process of wound healing (17), and their primary function is to construct and reshape the extracellular matrix. Fibroblasts are usually considered “immune neutral” cells, but further studies have revealed that fibroblasts play multiple roles in health and disease. In particular, fibroblasts have become the critical immune sentinel cells. They activate and regulate the immune response when detecting pathological stimulation as well as injury- and pathogen-related molecular patterns. They activate pro-inflammatory signaling pathways to help white blood cells recruit and regulate their activity (18–20). Thus, fibroblasts are now considered a “non-classical” branch of the innate immune system.

This review describes that fibroblasts may become an immunoregulatory factor in chronic inflammation and infection from the perspective of slow wound healing of diabetes, aiming to find the relationship between fibroblasts and the treatment of diabetic ulcers and provide new ideas for chronic refractory wound healing.

## Heterogeneity of fibroblasts

Diseases destroy normal cellular functions and cell–cell interactions and may lead to abnormal cell types and states, such as cancer cells. For more than 100 years, biologists have been trying to describe cell characteristics in increasing detail, including cell shape, location in tissues and relations with other cells, biological functions of cells, and molecular components, to identify the characteristics of cells and classify them into different types. However, it is only in recent years that the researchers’ systematic cell atlas seems to have begun to make it possible to carry out the systematic, high-resolution characterization of human cells (21). The first large-scale feasible single-cell genome characterization method is a transcriptional analysis by single-cell RNA sequencing (scRNA-seq) (22, 23). Nowadays, scRNA-seq can process and analyze tens of thousands of single cells simultaneously, reducing the measurement cost and improving the accuracy and sensitivity (24–26). scRNA-seq is rapidly spreading and changing our understanding of the pathogenesis of cross-medical diseases.

Researchers have found that fibroblasts are the key to wound healing through single-cell RNA sequencing. Experimentally, it has been shown that researchers have made scRNA-seq reanalysis on several samples, and it is found that fibroblasts in the wound are the key to hair follicle regeneration. Moreover, during the re-epithelialization of the wound, the papillary fibroblasts may migrate from around the wound to the center (27). At the single-cell level, scRNA-seq has enabled researchers to develop unexpected new insights into tissue biology and disease mechanisms by studying populations of cells in health and disease with unprecedented resolution.

### Healthy fibroblast heterogeneity

Fibroblasts are mesenchymal-derived cells that can produce collagen and extracellular matrix (ECM) proteins (28). As early as the 1960s, researchers noticed differences in the functional characteristics of fibroblasts from different tissues. With the gradual improvement of research methods, researchers have found that the heart (29), lung (30), gastrointestinal tract, and muscle (31) all contain fibroblasts with specific functions (32).

Epithelial cells from epicardial organs differentiate into mesenchymal stem cells (MSCs) through epithelial–mesenchymal transformation. Then, they differentiate into cardiac fibroblasts under the influence of many growth factors, such as platelet-derived growth factor (PDGF), fibroblast growth factor (FGF), and transforming growth factor (TGF) (33). Cardiac fibroblasts not only maintain the myocardial tissue structure and dynamic balance of ECM but also participate in the synthesis of connective tissue components and the production of factors related to the degradation balance, such as cytokines, growth factors, and matrix metalloproteinases (MMP), but may also passively affect electrical signals in the heart. Of note, recent research indicated that if electrically coupled with cardiomyocytes, they actively affect electrical signals in the heart (29). They illustrate the specific expression of discoid domain receptor 2 (DDR2) (34) and play a vital role in the regulation of normal myocardial function in addition to the pathological remodeling that accompanies hypertension and heart failure. It has long been recognized that the effects of the angiotensin II (Ang II) signaling pathway on cardiac fibroblasts and muscle cells lead to hypertrophy and fibrosis. Interleukin (IL)-1β and tumor necrosis factor-α (TNF-α) pro-inflammatory signals also lead to pathological changes in collagen synthesis of cardiac fibroblasts with concomitant myocardial failure (35).

Fibroblasts are also a component of lung structure. The effect of pro-inflammatory cytokines such as IL-1α on lung fibroblasts plays a crucial role in the mechanism of fibrotic lung disease (36). Axin2+/PDGFRα+ fibroblasts reside in the alveolar niche. Compared with other cell subsets, the fibroblast population has the effect of enhancing the self-renewal and differentiation of alveolar stem cells. Further studies have shown that FGF-7, IL-6, and the bone morphogenetic protein inhibitor gremlin 2 promote the self-renewal of this fibroblast population (37).

The distribution of fibroblasts in the intestinal tract is mainly customized according to the needs of the local tissue environment. Fibroblasts located in epithelial tissues such as the intestine specifically express SOX6 and periostin and exhibit transcriptional characteristics indicative of epithelial differentiation and cell proliferation to maintain barrier integrity and function (38, 39). In addition, the expression of components of the WNT pathway varied with their spatial locations. Specifically, fibroblasts closer to the villi expressed WNT5A and/or WNT5B, while fibroblasts in the lamina propria expressed WNT2B12. Conversely, synovial fibroblasts help maintain synovial health and joint lubrication in the absence of any epithelial cells. This is because synovial fibroblasts form a unique lining with resident macrophages (40).

**Figure 1:**
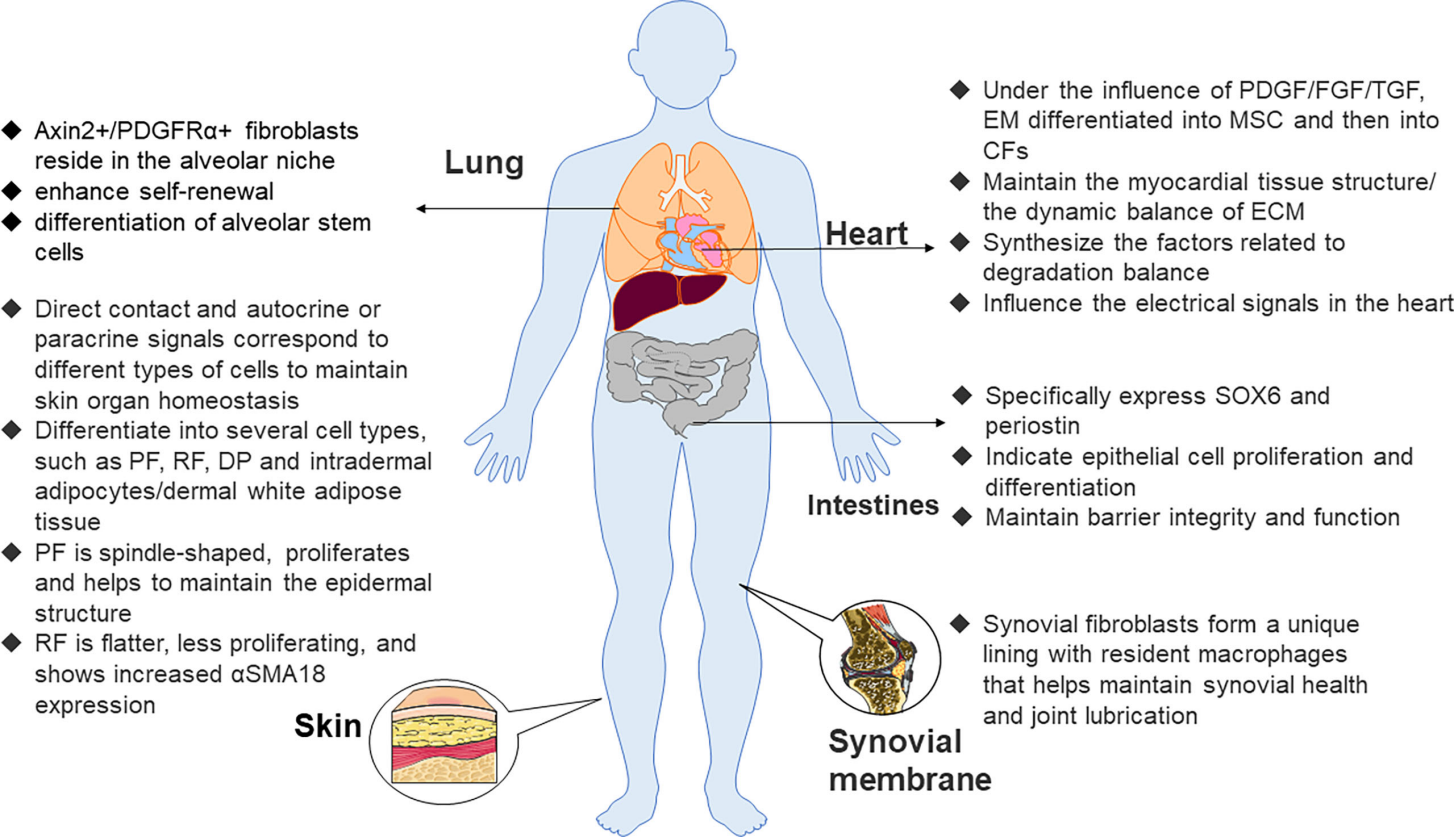
Functions of fibroblasts in various organs. Axin2, axis inhibition protein 2, PDGFRα, platelet–derived growth factor receptor α, PF, papillary fibroblast, RF, reticular fibroblast, DP, dermal papilla, α–SMA 18, α–smooth muscle actin 18, PDGF, platelet–derived growth factor, FGF, fibroblast growth factor, EM, epithelial mesenchymal, CF, cardiac fibroblast, MSC, mesenchymal stem cell, ECM, extracellular matrix.

Maintaining that steady state of skin organs requires continuous interaction between skin fibroblasts, keratinocytes, immune cells, nerve, and intradermal adipocytes (41, 42). Early embryonic fibroblast precursor cells may differentiate into several cell types, such as upper papillary fibroblasts (PFs), lower reticular fibroblasts (RFs), dermal aggregates, and intradermal adipocytes (43). As the upper PFs are spindle-shaped and proliferate and help maintain the epidermal structure, the lower RFs are flatter and proliferate less and prove to increase αSMA18 expression (44) (**Figure 1**) so that skin fibroblasts show a band boundary. The dermis is the connective layer between the epidermis and subcutaneous tissue, containing nerve endings, glands, blood vessels, and hair follicles, of which the most abundant cell type is the fibroblast. Dermal fibroblasts have multiple functions in the dermis, and they correspond to different types of cells through direct contact or autocrine and paracrine signals.

### Fibroblast heterogeneity in diabetes/diabetic wound healing

In clinically relevant studies on diabetes, organ dysfunction is usually associated with fibrotic changes. We have found myriad experimental studies on the direct fibrosis of diabetes and metabolic dysfunction (**Table 1**). Extensive investigation has shown that organs in patients with diabetes (e.g., kidney, heart, and liver) appear to be more prone to fibrous remodeling (55).

**Table 1:** Summary of experimental studies on diabetic fibrosis.

Source of fibroblasts	Protocol of glucose stimulation	Effects of glucose	Proposed mechanism		Ref.
The primary CFs of SD rat	Streptozotocin (STZ) injection strategy	Diabetic cardiac fibrosis and collagen deposition miR–21–3p was elevated AR was decreased	AR/caspase–1 pathway		(45)
The primary CFs of *db/db* mice	A point mutation	Decrease α–SMA Increase p–NF–κB expression	AGE/RAGE signaling		(46)
Human umbilical vein endothelial cells	High sugar culture cell *in vitro*	Upregulated expression of α–SMA/vimentin, downregulated expression of CD31/VE–cadherin, elevated transcription level of snail1/snail2/twist1/twist2	TGF–β/Smad signaling		(47)
H9C2 cardiomyocytes and primary CFs of C57BL/6J mice	High sugar culture cell *in vitro*	Upregulated collagen expression/apoptosis/overactive autophagy flux, FoxO1 nuclear translocation in cardiomyocytes Upregulated collagen/FoxO1 expression in CFs	Stimulation of AT4R, suppression of FoxO1 nuclear translocation, and inhibition of FoxO1–mediated overactive autophagy		(48)
Human renal tubular epithelial cells/rat kidney fibroblasts/male Sprague–Dawley rats	High sugar culture cell *in vitro*/STZ injection strategy	Induce ECM accumulation of renal tubular epithelial cells and renal interstitial fibroblasts	miR–192–5p/GLP–1R pathway		(49)
Rat mesangial cells	High glucose culture	circ–ITCH under expression Inhibited the viability/migration/fibrosis/inflammatory response of RMCs	The miR–33a–5p/SIRT6 axis		(50)
Primary liver cells of C57BL/6J mice/LX–2 cells	TGF–β1	Induce the massive accumulation of ECM components, expression of α–SMA, and cell proliferation/secrete numerous pro–fibrogenic cytokines	PTEN/AKT pathway		(51)
Sprague–Dawley rats	STZ injection strategy	ALT, AST, and ALP levels were increased, TG and GGT levels induced, infiltration of inflammatory cells in the hepatic portal area, hyperplasia of fibers and the bile duct			(52)
Human MIO–M1 Müller cells	Notch ligands and TGF–β1	ECM protein overexpression	Notch signaling		(53)
Human monocytic THP–1 cell/human dermal fibroblast/C57/BL6J	High glucose culture/STZ	Macrophage polarization decreased, HDF proliferation and migration decreased, wound healing was slowed down and inflammation worsened. Less vascular regeneration and reduced IL–25 expression	PI3K/AKT/mTOR and TGF–β/Smad signaling	(54)	

Significant diversity and functional heterogeneity have been found during organ fibrosis within the fibroblast population (56). In cardiovascular disease, fibroblasts play a central and dynamic role in myocardial remodeling. Fibroblast proliferation and migration, and even the extent and composition of the cardiac ECM, are affected (57). The development of cardiac fibrosis is often recorded in patients with advanced diabetes (58). Diabetic myocardial fibrosis involves the effects of hyperglycemia, lipid toxicity, and insulin resistance on cardiac fibroblasts, which directly leads to the increase of matrix secretion and activates the paracrine signals in myocardial cells, immune cells, and vascular cells (57). Hyperglycemia leads to the accumulation of AGEs, which in turn conduct fibrotic signals through reactive oxygen species or through a pathway mediated by the activation of the RAGE. The TGF-β/Smad signaling pathway may also activate fibroblasts. Pro-inflammatory cytokines and chemokines recruit fibrotic white blood cell subsets, which indirectly mediate cellular fibrosis (59–61).

Furthermore, studies have revealed that fibrous tissue remodeling is associated with increased expressions of MMP and humoral factors such as TGF, Ang II, endothelin-1 (ET-1), and TNF-α (29, 62). Hyperglycemia is a central stimulating factor that activates the progression of diabetic renal fibrosis (63). Hence, several lines of investigation suggest that in patients with diabetes, fibrosis changes are associated with increased renal insufficiency (64, 65). Researchers used medications that inhibit neurohumoral fibrosis by delaying renal fibrosis in people with diabetes (66) and thus reduce renal insufficiency.

Patients with diabetes may develop non-alcoholic fatty liver disease (NAFLD), a disease associated with hepatic steatosis (67–69). Oxidative stress and macrophage-driven inflammation activate hepatic stellate cells and promote their transformation into synthetic stromal myofibroblasts (70). Hepatic fibrosis in patients with NAFLD can be improved when anti-diabetic drugs such as metformin or thiazolidinediones are administered. Significant weight loss in severely obese NAFLD patients is also helpful to improve hepatic fibrosis (71, 72).

**Figure 2:**
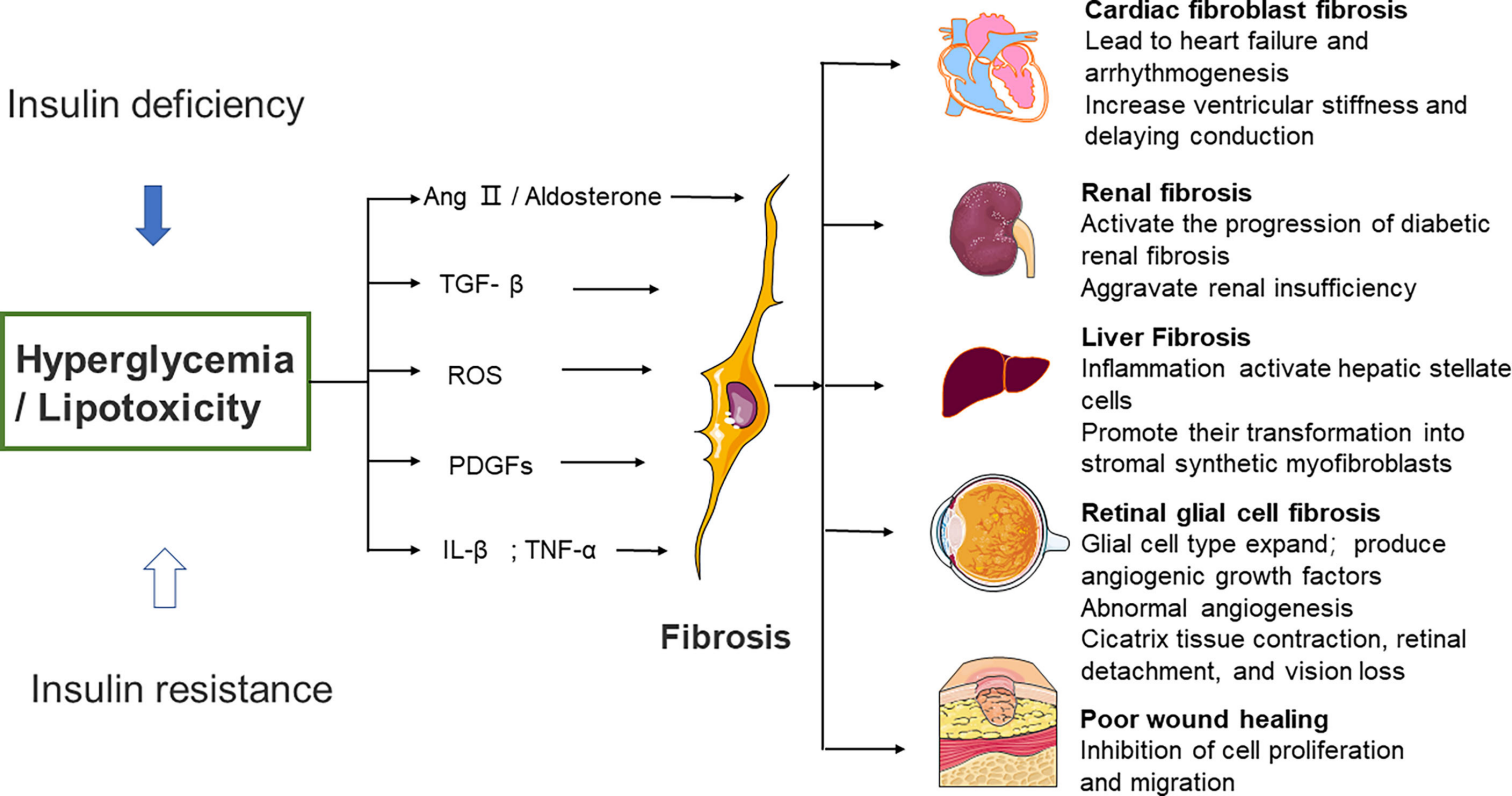
Effects of high glucose on fibroblast activity and fibrotic function of different organs. Several different mechanisms of diabetic fibrosis: 1) the activation of neurohumoral pathways, 2) induction and activation of growth factors such as TGF–β and platelet–derived growth factors, 3) stimulation of pro–inflammatory cytokines such as TNF–α and IL–1β. The main pathway that fibroblasts activate in response to high glucose involves ROS production, 4) high glucose can aggravate the damage of ROS to cells, promote cell senescence, and cause chronic inflammation and fibrosis. Ang II, angiotensin II, ROS, reactive oxygen species, PDGFs, platelet–derived growth factors, IL–β, interleukin–β, TNF–α, tumor necrosis factor α.

Diabetic patients develop retinopathy. Müller cells (the predominant glial cell type in the retina), astrocytes, and microglia expand to obtain a fibroblast-like phenotype, which then produces vascular growth factors and secretes ECM proteins (73, 74). Fibrovascular proliferation is characterized by abnormal angiogenesis and excessive matrix deposition. This is mediated by glial cells and causes scar tissue shrinkage, retinal detachment, and vision loss (75). Poor wound healing in diabetes is associated with impaired myofibroblast function. Researchers observed decreased expression of fibrosis-related genes and decreased expression of fibrosis-related genes in diabetic wounds 5 days after injury, compared to myofibroblasts from db/+ mouse wounds (76) (**Figure 2**).

## Immune regulation of fibroblasts

Fibroblasts act as guardians of the immune balance by controlling the recruitment, activation, and removal of immune cells, shifting the immune system from a controlled immune state to a sustained, irreversible inflammatory or immunosuppressive environment. Therefore, it is imperative to deeply study how fibroblasts regulate the immune response to inflammation and infection.

### Immune regulation in inflammatory processes

The resolution of inflammation is pivotal for wound healing and ECM formation. For a long time, chronic inflammation in diabetic patients is also a monumental reason for the long-term non-healing of diabetic wounds. A growing body of scientific evidence strongly suggests that the poor healing of diabetic wounds is associated with dysregulated fibroblast to myofibroblast differentiation, disrupted myofibroblast activity, and insufficient extracellular matrix production (77). By intradermal injection of interleukin-25 into mice, researchers found that M2 macrophages increased polarization, which improved wound angiogenesis and led to collagen accumulation. The results showed that IL-25 could regulate the function of fibroblasts and promote the activation of fibroblasts in a high glucose environment (54). Another study has shown increased secretion of fibroblast IL-6 in diabetic donors (78). Nowadays, there is abundant evidence supporting the capability of non-immune cells to coordinate inflammation in diseases, and fibroblasts can be involved in pro-inflammatory signaling after injury (79).

The role of fibroblasts in primary lymphoid organs and secondary lymphoid organ (SLO) has long been recognized, and the importance of fibroblast–immune cell interaction in peripheral tissues has also received increasing attention (80). Lymphoid tissue controls the proper differentiation and release of circulating leukocytes and enables the convergence and transformation of innate and adaptive immune cells (81). In addition, extensive study data indicate that bone marrow fibroblasts are capable of inhibiting immature cells until they differentiate and properly disperse into the blood. This is mediated by CXC chemokine ligand 12 (CXCL12) and vascular cell adhesion protein 1. Moreover, the researchers also found that bone marrow fibroblasts promote B-cell maturation, mediated by a single group that produces IL-7 (82).

The SLO, composed of lymph nodes (LNs), spleen, and peyer's patches (PPs), is the site of immune cell monitoring of the body. SLO maintains immune homeostasis and promotes a rapid and effective immune response (83). Tissue-derived dendritic cells enter the LNs through the afferent lymphatic vessels, lymphocytes enter through the HEV, and their migration to their unique niches is supported by highly specialized fibroblast matrix cells. Collectively referred to as fibroblast reticular cells (FRCs), these cells comprise subsets such as the T-cell region (FRC or TRC), follicular dendritic cells (FDCs), marginal reticular cells (MRCs), and medullary FRC (medRC). In fact, recent evidence suggests the presence of additional FRC subsets in LNs and other SLOs. Their developmental origins, markers, and exact functions are areas of future research (84).

Through scRNA-seq analysis, the researchers found that specific anatomical locations and their interaction with immune cells determine the identity of FRC subsets, and phenotypic differences of FRC subsets are classified and illustrated (85, 86). T-cell zone reticular cells are located beside the cortex. *In-vitro* experiments showed that T-zone fibroblast reticular cells could recruit, nest, and maintain immature T cells by secreting IL-7 and the CCR7 ligand CCL19. As such, several studies have displayed that since the LNs and the T-zone FRCs provide a limited pool of survival factors, they can play a crucial role in T-cell homeostasis (87). Reticular cells that interact with B cells, including follicular dendritic cells from germinal centers, produce CXCL13 to recruit and maintain a pool of immature B cells (88).

There is also a growing body of evidence to indicate that marginal reticular cells are located between the subcapsular sinus and the B-cell follicles. The LN follicular dendritic cell (FDC) network is produced by clonal expansion and differentiation of MRCs (89).

Importantly, FRCs also promote tolerance to autoantigens and regulate the immune response against commensal bacteria through various mechanisms. It involves the activation of regulatory T cells, and these findings illustrate that FRCs inhibit T-cell activation by producing high levels of prostaglandin E2 (PGE2) during homeostasis, as their cyclooxygenase-2 (COX-2) expression is thousands of times that of immune cells. The inhibition of this overactive COX-2/PGE2 production was evident during antigen-specific and non-antigen-specific activation (90, 91). In addition, FRC can present antigens directly to T and B cells, activating an adaptive immune response and deleting or inducing dysfunction of self-reactive lymphocytes (92, 93). Thus, fibroblasts in lymphoid tissue regulate innate lymphocytes and myeloid cells and recruit, activate, and retain lymphocytes. However, there is also an immunosuppressive mechanism for fibroblasts in order to maintain balance in the body.

### Constituent of tertiary lymphoid tissue

Tertiary lymphoid structure (TLS) is associated with cancer, infection, and autoimmunity (94). TLS is an organized cluster of lymphocytes capable of supporting an antigen-specific immune response in non-immune organs. These lymphoid nascent ectopic sites consisted of an organized structure of lymphocytes (B cells and immature T cells), myeloid cells (LAMP3+ DC), and stromal cells. Researchers observed structures like secondary lymphoid tissues in different regions of chronic inflammation, suggesting that chronic inflammation and lymphoid tissue formation share a common activation process. Ectopic lymphomagenesis occurs similarly to SLOs, which relies on the passage of the lymphotoxin-β receptor (LtβR; also known as TNFRSF3) signal path (95).

**Figure 3:**
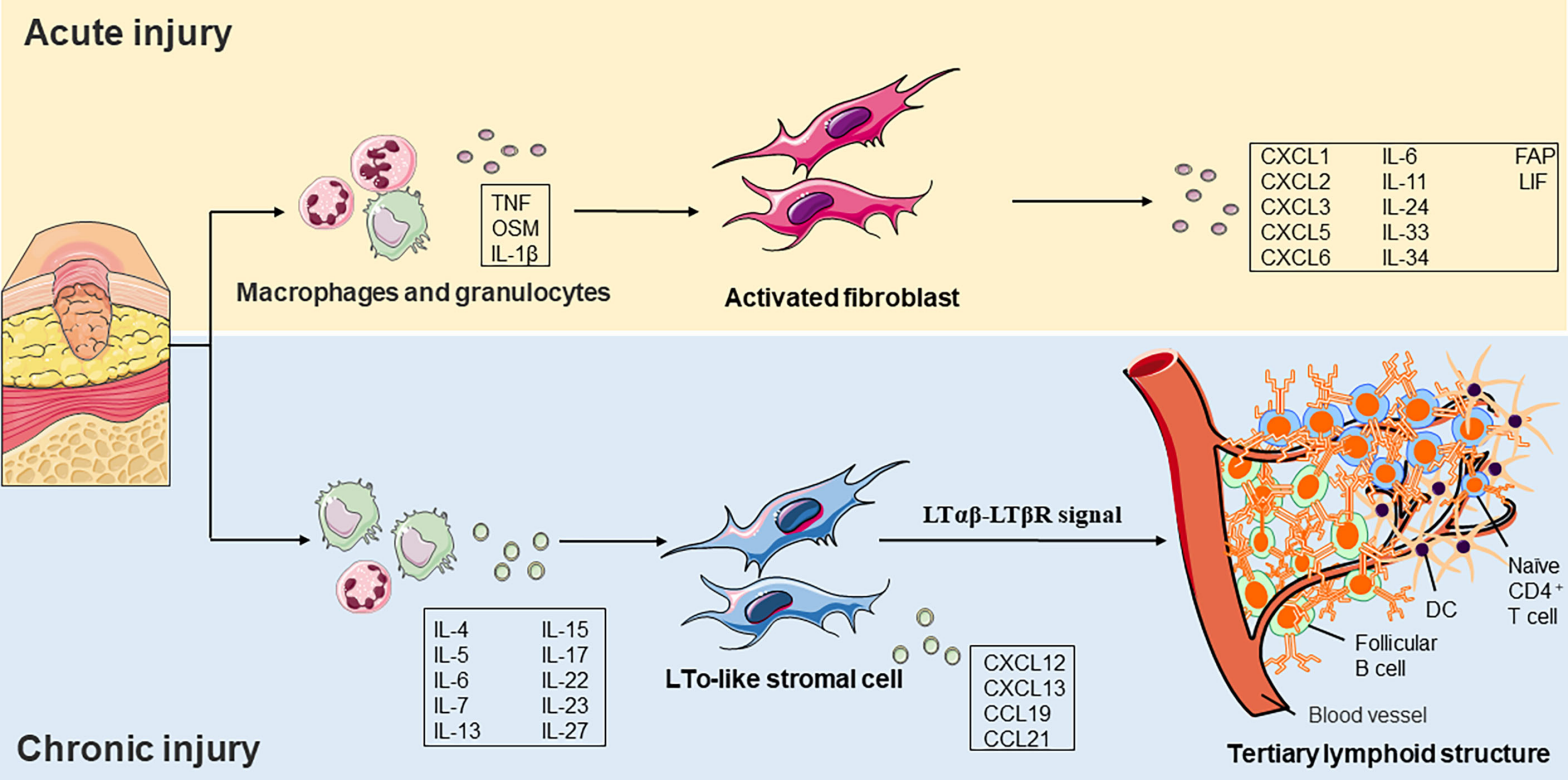
Role of fibroblast functions in acute and chronic injury. a) In acute injury, tissue damage activates pathogen– and damage–related molecular patterns on macrophages and granulocytes. Cytokines subsequently released by these cells, such as TNF or OSM, and IL–1β induce a pro–inflammatory phenotype fibroblast formation. b) The local cytokine environment initiates LTo–like stromal cells in a chronic inflammatory environment. It induces the expression of chemokines such as CCL19, CCL21, CXCL12, and CXCL13. LTo–like stromal cells recruit immature CD4+ T cells, LAMP3+ DCs, and follicular B cells and eventually form a tertiary lymphoid structure. TNF, tumor necrosis factor, OSM, oncostatin M, IL, interleukin, LTo, lymphoid tissue organizer, CCL, CC–chemokine ligand, CXCL, CXC–chemokine ligand, DC, dendritic cell, FAP, fibroblast activation protein, LIF, leukemia inhibitory factor, LTβR, lymphotoxin–β receptor.

Accumulating shreds of evidence have indicated that molecular mediators present in the chronic inflammatory environment can promote the initiation of TLS development. The plasticity and specialization of fibroblasts under inflammatory conditions have recently been revealed in immune and non-immune organs. In addition to recruiting and inhibiting leukocytes, fibroblasts also promote TLS formation and then maintain immune permanence (96). Researchers have demonstrated that in TLS, resident fibroblasts expand and acquire immune characteristics in a process that is dependent on IL-13 and IL-22. Interference with the expansion of resident fibroblasts or depletion of immune fibroblasts leads to TLS degradation, further reducing immune cell activation (97) (**Figure 3**). There are still many debates on the function and clinical significance of TLS in diseases. Several lines of evidence suggest that CXCL13 and ectopic lymphomagenesis are drivers of inflammation and associated with adverse clinical outcomes, but others have questioned this hypothesis (98, 99). The skin ulcer wound of diabetic patients is in chronic inflammation for a long time, and whether TLS can improve the poor healing of the ulcer wound is the direction of our subsequent fundamental research.

### Coordinated immune response during infection

Chronic wounds were repeatedly infected and showed persistent abnormal inflammation. The process of re-epithelization of the wound was stopped, but the keratinocytes were excessively proliferated. Overexpression of MMPs, poor fibroblast infiltration, and slow angiogenesis are all causes of wound failure to heal (100). It is found that virus infection will reprogram FRC characteristics to guide the migration and differentiation of innate and adaptive immune cells. FCR in LNs of infected sites profoundly changed the expression patterns of genes involved in antigen presentation, ECM remodeling, and chemokine and cytokine signal transduction (101).

IL-17-producing T helper (TH17) cells promote inflammation by inducing cytokines and chemokines in peripheral tissues. Researchers demonstrate a critical requirement for IL-17 in the proliferation of LN and spleen stromal cells, particularly FRCs (102).

In addition to the role of FRCs in SLOs–functional tissues during the infection process, fibroblasts resident in tissues can also directly respond to microbial signals to play physiological functions. TNFSF15-mediated fibroblast activation and conversion to myofibroblasts in chronic inflammation and fibrosis of the intestinal tract may require specific microorganisms (103). Therefore, the extent of inflammation-induced transcriptional remodeling reflects the dynamic nature of FRC immune cell interaction, which changes over time, indicating the adaptability of a particular immune environment to different pathogens.

## Conclusion and prospect

Increasing scientific evidence from existing experimental studies in animal and cellular models strongly correlates wound healing disorders in diabetic patients with dysfunctional fibroblast differentiation, disrupted myofibroblast activity, and inadequate extracellular matrix production. The results exhibited that high glucose had a detrimental effect on the proliferation and migration of hepatocyte growth factors (HGFs). Oxidative stress in a high glucose environment is the cause of fibroblast dysfunction, which delays gingival wound healing in diabetic patients (104). In addition, experimental data showed a significant reduction in myofibroblasts on wound days 5 and 7 in diabetic rats compared to non-diabetic rats. Diabetic wound healing has been associated with decreased epithelial and connective tissue remodeling in early diabetic periodontal wounds, increased levels of inflammation, and delayed myofibroblast differentiation. On the basis of these findings, hyperglycemia can interfere with cytokine signaling pathways that affect fibroblast differentiation (such as the growth factor-β pathway), change fibroblast apoptosis, regulate dermal lipolysis, and enhance hypoxic injury, resulting in a damaged microenvironment for myofibroblast formation, improper regulation of extracellular matrix, and weakened wound contraction (77, 105).

However, excessively activated fibroblasts can lead to fibrosis. Diabetes–related morbidity and mortality are caused by complications that may lead to organ failure conditions such as heart and kidney failure, hepatic insufficiency, retinopathy, or peripheral neuropathy. Excessive and inappropriate deposition of fibroblasts in various tissues may lead to organ dysfunction, commonly in advanced type 1 or type 2 diabetes patients. Hyperglycemia, lipotoxic damage, and insulin resistance activate fibrotic responses by directly stimulating matrix synthesis by fibroblasts and possibly by promoting fibrotic phenotypes in immune and vascular cells triggering epithelial and endothelial cell transformation to a fibroblast–like phenotype.

The multiple phenotypes of fibroblasts lead to their very complex functions. During embryogenesis, fibroblasts reside in tissues and form sentinel cells according to the needs of the surrounding tissues through epigenetic imprinting. When exposed to injury, they help initiate, control, and moderate a subsequent immune response. For example, it can interact with granulocytes and bone marrow cells and collect and retain lymphocytes. Especially in the case of infection, it appears as an immune outpost cell in the form of TLS.

In the chronic inflammation of diabetes, insufficient differentiation of early fibroblasts leads to poor wound healing. However, inappropriate fibroblast activation induces pro–inflammatory and immunosuppressive properties that promote disease persistence, but fibrosis leads to severe organ dysfunction in the advanced stage of diabetes. Then, further in–depth study of fibroblasts may help restore homeostasis in the disease. Therefore, studying the functional and dynamic changes of fibroblasts in chronic skin ulcers and releasing their therapeutic potential in tissue repair are the research directions in the future.

## Author contributions

YL was responsible for the literature review and writing. YQL, WH, XM, XW, and JD were responsible for the correction. XN was responsible for the proofreading, literature review, and correction. All authors contributed to the article and approved the submitted version.

## Funding

This work was supported by the National Natural Science Foundation of China (81960741, 82160770), the Guizhou Provincial Natural Science Foundation (QKH–J–2020–1Z070), the Outstanding Young Scientific and Technological Talents Project of Guizhou Province (2021–5639), and the Dendrobium Specialized Class Project of Guizhou Province (QSKH–2019003).

## Conflict of interest

The authors declare that the research was conducted in the absence of any commercial or financial relationships that could be construed as a potential conflict of interest.

## Publisher’s note

All claims expressed in this article are solely those of the authors and do not necessarily represent those of their affiliated organizations, or those of the publisher, the editors and the reviewers. Any product that may be evaluated in this article, or claim that may be made by its manufacturer, is not guaranteed or endorsed by the publisher.
